# Nodeomics: Pathogen Detection in Vertebrate Lymph Nodes Using Meta-Transcriptomics

**DOI:** 10.1371/journal.pone.0013432

**Published:** 2010-10-18

**Authors:** Nicola E. Wittekindt, Abinash Padhi, Stephan C. Schuster, Ji Qi, Fangqing Zhao, Lynn P. Tomsho, Lindsay R. Kasson, Michael Packard, Paul Cross, Mary Poss

**Affiliations:** 1 Department of Biochemistry and Molecular Biology, Center for Comparative Genomics and Bioinformatics, The Pennsylvania State University, University Park, Pennsylvania, United States of America; 2 Department of Biology, Center for Infectious Disease Dynamics, The Pennsylvania State University, University Park, Pennsylvania, United States of America; 3 Northern Rocky Mountain Science Center, U.S. Geological Survey, Bozeman, Montana, United States of America; 4 Fogarty International Center, National Institutes of Health, Bethesda, Maryland, United States of America; Duke University Medical Center, United States of America

## Abstract

The ongoing emergence of human infections originating from wildlife highlights the need for better knowledge of the microbial community in wildlife species where traditional diagnostic approaches are limited. Here we evaluate the microbial biota in healthy mule deer (*Odocoileus hemionus*) by analyses of lymph node meta-transcriptomes. cDNA libraries from five individuals and two pools of samples were prepared from retropharyngeal lymph node RNA enriched for polyadenylated RNA and sequenced using Roche-454 Life Sciences technology. Protein-coding and 16S ribosomal RNA (rRNA) sequences were taxonomically profiled using protein and rRNA specific databases. Representatives of all bacterial phyla were detected in the seven libraries based on protein-coding transcripts indicating that viable microbiota were present in lymph nodes. Residents of skin and rumen, and those ubiquitous in mule deer habitat dominated classifiable bacterial species. Based on detection of both rRNA and protein-coding transcripts, we identified two new proteobacterial species; a *Helicobacter* closely related to *Helicobacter cetorum* in the *Helicobacter pylori/Helicobacter acinonychis* complex and an *Acinetobacter* related to *Acinetobacter schindleri.* Among viruses, a novel gamma retrovirus and other members of the Poxviridae and Retroviridae were identified. We additionally evaluated bacterial diversity by amplicon sequencing the hypervariable V6 region of 16S rRNA and demonstrate that overall taxonomic diversity is higher with the meta-transcriptomic approach. These data provide the most complete picture to date of the microbial diversity within a wildlife host. Our research advances the use of meta-transcriptomics to study microbiota in wildlife tissues, which will facilitate detection of novel organisms with pathogenic potential to human and animals.

## Introduction

Information about the commensal and pathogenic microbial communities associated with host species, including humans, is limited. The endemic microbial community of a healthy host is important to characterize because its perturbation can be a cause of disease [1], [2]. Pathogenic microbes often escape detection if the clinical consequences of infection are similar to known pathogens or if they infect non-domestic species [3]. The maintenance of unknown pathogens in wildlife species is particularly problematic because many emerging human and livestock infections arise from contact with wild animals [4]–[7].

With the advent of meta-genomics methods, the entire community of microorganisms that exist in a given environment can potentially be identified. Pyrosequencing and other high throughput sequencing approaches have been applied to determine the microbial population in environmental samples such as soil and seawater [8]–[11] and more recently to investigate the community of microbes on human mucosal surfaces [12]–[15], both of which are rich in microorganisms. Next generation sequencing methods have also been successfully applied to identify the microbial agents of several new diseases [16]–[20]. Recently, RNA based meta-transcriptomic studies [21]–[23], which profile both protein-coding transcripts and ribosomal RNA (rRNA), have been used to study both functional and structural features of environmental microbial communities.

The key question behind this study was whether viable microorganisms could be detected within healthy mammalian lymphoid organs by employing massively parallel sequencing coupled with computational techniques able to detect transcripts of microorganisms among the abundant transcripts of the mule deer host. Lymph nodes are the specific replication sites for certain pathogenic viruses and bacteria [24]–[29]. Moreover, although the blood and the lymph systems are considered to be essentially free of viable microorganisms in healthy individuals, the transient and often asymptomatic presence of both bacteria and viruses have been detected in the circulation [30], [31]. Phagocytic cells engulf these microbes and migrate to lymph nodes. Thus, lymph nodes should concentrate the commensal, endemic, and potential pathogenic microbial communities of a host species.

We evaluated the microbial community in retropharyngeal lymph nodes of mule deer to assess microbial exposure via the oral or respiratory route. Because ungulates browse and receive small punctures from sharp forage, we reasoned that healthy animals would potentially be exposed to microorganisms from their environment or to resident oral and rumen microorganisms that would be cleared in draining nodes. We used mule deer to highlight the utility of this approach in a wildlife host, but the method is broadly applicable to any host species.

Our studies document for the first time that there is a community of viable microorganisms in retropharyngeal lymph nodes of healthy wild ungulates. Furthermore, our findings demonstrate the applicability of meta-transcriptomic techniques for the detection of novel bacteria and viruses in internal organs.

## Results

### The microbial community of mule deer lymph nodes

**Figure 1:**
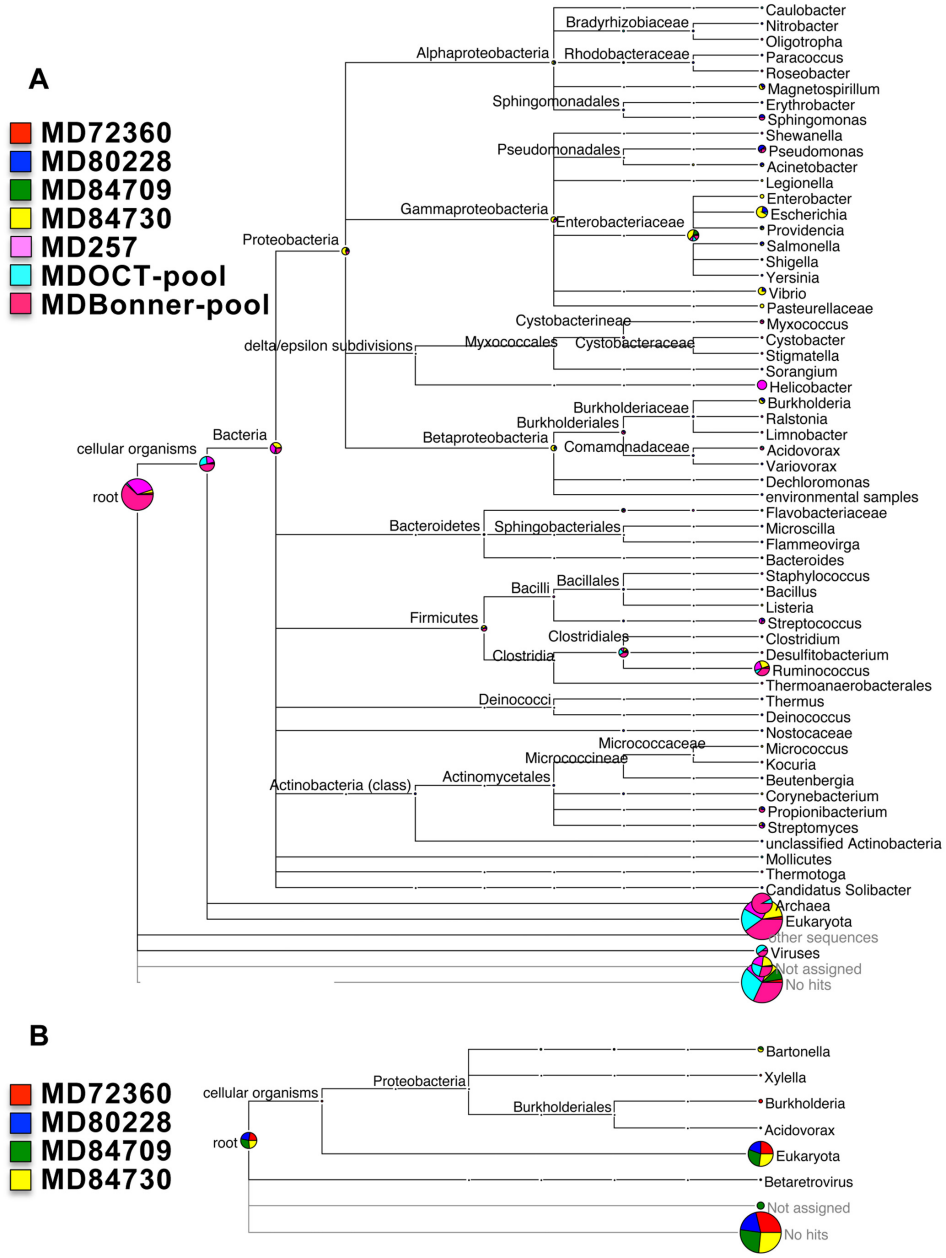
MEGAN comparison of the taxonomic profiles of (A) cDNA transcript-tags from 454 sequencing five individual lymph node samples and two lymph node sample pools and (B) genomic DNA-tags from four individual lymph node samples. Depicted are assignments with bit score cutoffs ≥50. Circle sizes are scaled logarithmically. Not assigned: sequencing-tags matching to sequences in the NCBI database that are not assigned to taxa; no hits: sequencing-tags not matching to any sequences in the NCBI database.

Detection of protein-coding and ribosomal RNA transcripts provides strong support for the presence of viable and replicating microorganisms. Therefore, we enriched the total RNA obtained from lymph nodes for poly(A)^+^ RNA to prepare cDNA libraries and subjected them to pyrosequencing on a Roche GS FLX sequencer (Roche-454 Life Sciences). Properties of sequencing runs are given in Table S1. All reads were compared against the nonredundant NCBI protein database. The composite meta-transcriptomic species profile for five individual and two pools of 4 or 8 mule deer samples, determined using the software MEGAN [32], is depicted in Fig. 1A.

On average, 51% of total transcript-tags could be assigned to known taxa with a bit score cutoff of 50 (see Table S2). Of the assigned tags, 99.3% were of eukaryotic origin, predominantly matching to *Bos taurus* and other close relatives of mule deer that are represented in the protein database. Approximately 0.3% of the assigned tags were to bacteria. Proteobacteria represented 60% of all bacterial hits; Enterobacteriaceae in the Gammaproteobacteria were the most commonly identified within this group. Firmicutes and Actinobacteria represented 22% and 5% of the identified bacterial taxa. Table S3 lists all bacterial genera detected in the seven data sets. Transcripts assigned to Archaea, family Halobacteriaceae, were identified in both pooled samples but none of the individual libraries. Only 37 transcripts were assigned to viruses. Twenty-nine of these matched to the Retroviridae and Poxviridae while the remaining were to phages, insect viruses, and a single assignment to herpesvirus. These results suggest that representatives of many bacterial phyla, archaea, and two major virus families are transcriptionally active in mule deer retropharyngeal lymph nodes.

Meta-genomics studies evaluating microbial rich communities were pioneered based on genomic DNA sequences [8]–[10], [13]. Thus, we compared genomic libraries prepared from retropharyngeal lymph node tissue of MD 72360, MD 80228, MD 84709, and MD 84730 with our data from transcript libraries derived from those animals (Fig. 1). Many sequences from the genomic DNA libraries were to non-coding regions and could not be used for taxonomic profiling (Fig 1B, Table S2). Based on protein-coding sequences, only four bacterial genera were identified in the comprehensive MEGAN analysis of the four genomic data sets. *Xylella* and *Burkholderia* were identified in MD 72360, *Acidovorax* was found in MD 84709, and *Bartonella* was found in both MD 84709 and MD 84730. *Bartonella* and *Xylella*, as well as a member of the beta retroviruses (found in MD 80228 and MD 84709), were identified only in the genomic DNA data, suggesting that they might not represent actively replicating organisms. These findings indicate that meta-transcriptomics may be the preferred method for detecting the viable endemic microbial community in the tissues of healthy animals.

The most commonly detected microorganisms in the transcriptome libraries comprised intestinal and skin-dwelling bacteria and soil and freshwater bacteria. *Ruminococcus*, which is part of the commensal intestinal microbial community of ungulates, was detected in all seven libraries (Fig. 1A and Table S3). Other bacteria found in at least three of the seven data sets were *Propionibacterium*, a commensal bacterium of skin and the gastrointestinal tract, and the environmental soil or water inhabitants *Magnetospirillum*, *Streptomyces* and *Pseudomonas*. Members of the latter genus are able to colonize a wide range of niches and are also potential pathogens. Other animal and human pathogenic genera detected in at least three different libraries were *Burkholderia*, *Streptococcus*, *Flavobacteria*, and members of the Enterobacteriaceae (*Escherichia*, *Providencia*).

The overall bacterial diversity and the number of unique transcripts assigned to each bacterial taxon varied among the samples. Notably, *Helicobacter* was only detected in the library constructed from MD 257 but there were 12 unique transcript-tags assigned to this genus. More commonly, bacterial taxon identification was based on a single tag. Many of the single transcript-tags came from MD 80228, which had the highest bacterial diversity profile of all libraries analyzed, and from MD 84730. Bacterial genera detected solely in either one or both of these two samples include *Acinetobacter*, *Legionella*, *Enterobacter*, *Salmonella*, *Yersinia*, *Vibrio*, *Listeria*, *Mannheimia,* and members of the Corynebacterineae, all of which contain known pathogens. In addition, both specimens depicted by far the highest numbers of reads taxonomically assigned to the family Enterobacteriaceae. The lowest diversity of bacterial genera was found in the MD OCT-pool, which was derived from eight different mule deer. Pooling RNA from several animals potentially increases the representation of transcripts common to all animals but might decrease the ability to detect transcripts that are unique to one animal. Consistent with this, the MD Bonner-pool, which was derived from four animals, provided a broader spectrum of bacterial genera than the MD OCT-pool. Thus, pooling samples did not improve our ability to detect microbial diversity in lymph node samples.

In contrast, viruses were detected in both pooled samples, although the total number of transcript-tags was low. Of the individual libraries, only MD 257 had evidence of viral transcripts (Fig. 1A). The majority of viral transcripts were from a cervid poxvirus [33], and a novel gamma retrovirus.

### Identification of novel microorganisms

**Figure 2:**
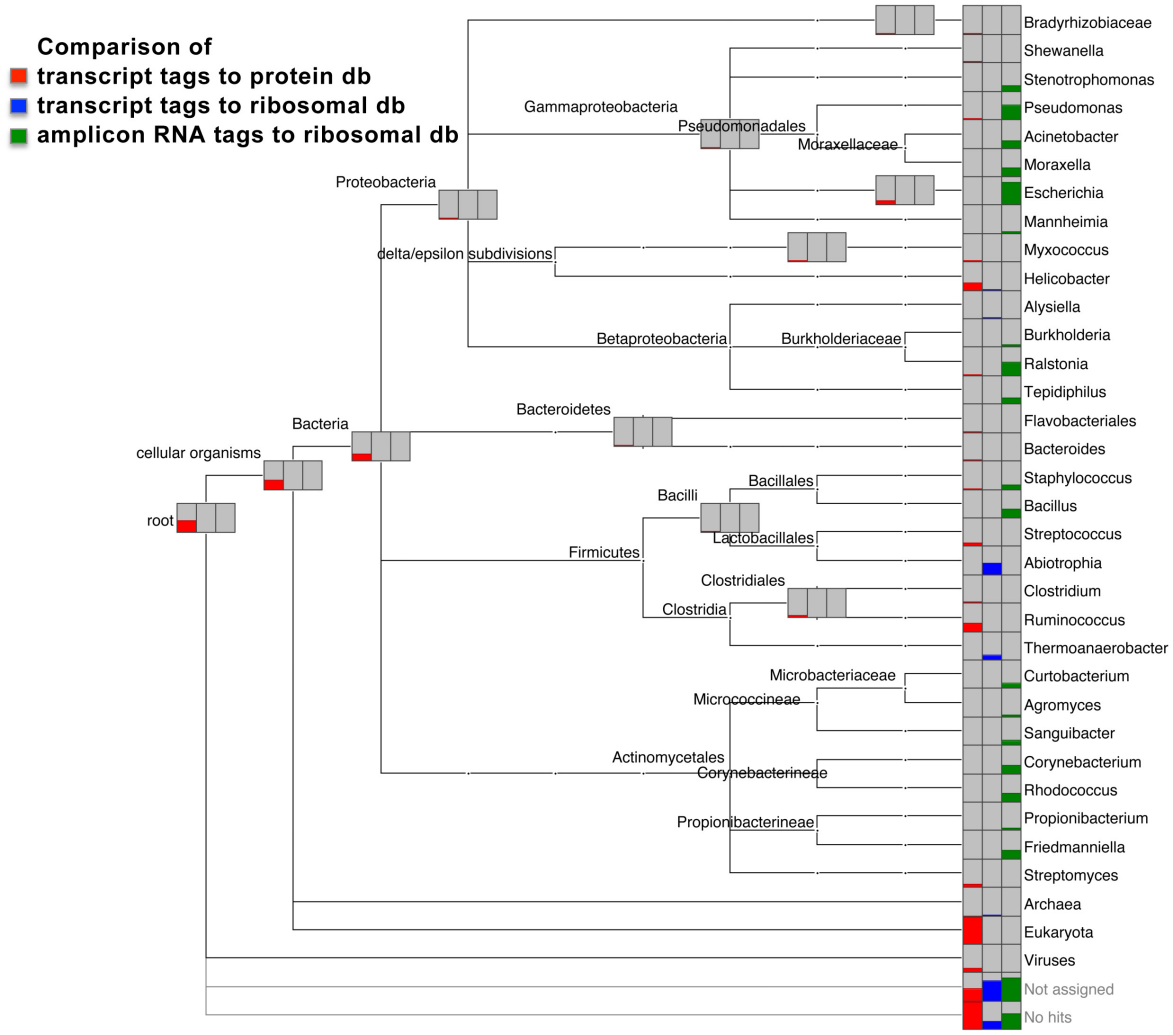
MEGAN comparison of taxonomic profiles of MD 257 cDNA transcript-tags analyzed against the protein database (red) and the ribosomal database (blue), and of V6 amplicon 16S rRNA-tags analyzed against the ribosomal database (green). Bit score cutoff for the protein database comparison was set at 50, and confidence cutoffs for the ribosomal database comparisons were set at 80%.

The computational analysis described above identified putative microorganisms in mule deer tissue based on detection of protein-encoding transcriptional activity. Although the cDNA used in our analyses was derived from total RNA enriched for polyadenylated RNA, it retained a considerable amount of the abundant ribosomal RNA (rRNA). These sequences contribute to the ‘no hits’ category in Figure 1 and Table S2. Bacterial rRNA derived from the same dataset can, therefore, be used to provide additional support for species identification. By classifying the rRNA-tags from each library using the RDP rRNA classifier tool [34], [35] (http://rdp.cme.msu.edu/) we increased the number of bacterial genera identified (see Fig. 2 for MD 257, Fig. S1 for MD 80228 and MD OCT-pool, and Table S4). *Abiotrophia*, which is a component of the normal oral and intestinal microbial community, was detected in six of the seven libraries; environmental bacteria such as *Thermoanaerobacter*, which is frequently found in hot springs, were detected in four of the seven samples. Other genera that were identified based on rRNA in at least two of the libraries were *Actinomyces*, *Campylobacter* and *Mycoplasma*. Of particular importance, rRNA-tags supported the presence of *Helicobacter* in the MD 257 library (Fig. 2), of *Acinetobacter*, *Escherichia*, *Pseudomonas*, *Salmonella*, *Shigella* and *Variovorax* in the MD 80228 library, and of *Shigella* in the MD Bonner-pool library (Fig. 1 and Fig. S1, Tables S3 and S4).

**Figure 3:**
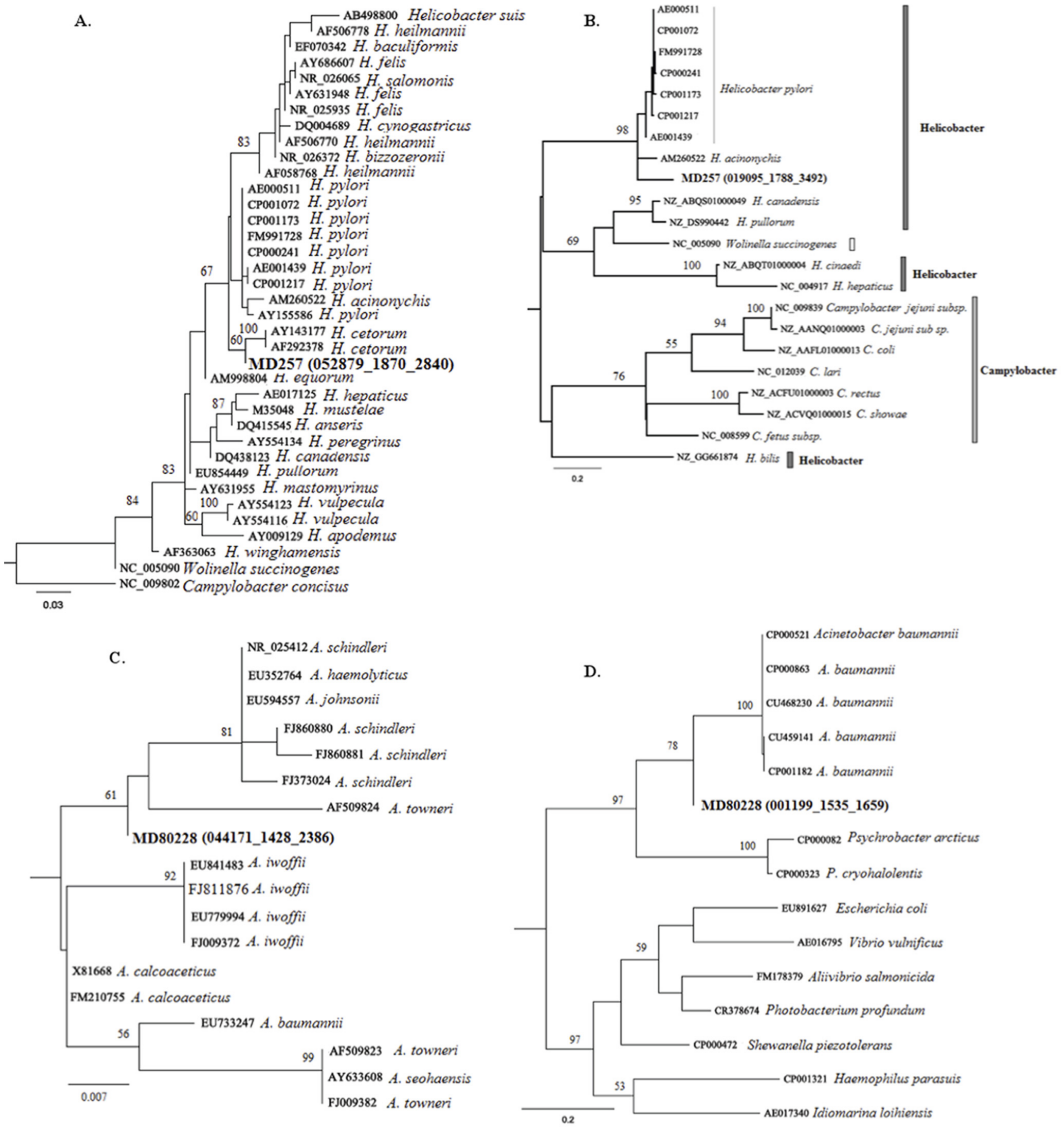
Maximum likelihood trees showing the phylogenetic affiliation of sequences obtained from 454 sequencing with GenBank homologous sequences. (A) 16S rRNA *Helicobacter*, (B) *rpo-*β *Helicobacter,* (C) 16S rRNA *Acinetobacter*, (D) *rpo-*β *Acinetobacter*. Bootstrap support for each node is indicated.

The support for *Helicobacter* in the MD 257 library was particularly compelling because there were 12 unique transcript-tags and one rRNA-tag to this genus. We evaluated the phylogenetic relatedness of the mule deer *Helicobacter* with other *Helicobacter* based on four of the protein-coding transcripts and on the single 16S rRNA sequence. All analyses demonstrated that the *Helicobacter* detected in the mule deer lymph node is a unique organism that affiliates with the *H. pylori* cluster (Fig. 3A and 3B, and Fig. S2). Because 16S rRNA sequence data is available for more species, we were able to further demonstrate that the closest relative to mule deer *Helicobacter* is a newly described *H. cetorum* isolated from different dolphin species (*Lagenorhynchus acutus*, *Lagenorhynchus obliquidens*, and *Tursiops truncatus*) and a beluga whale (*Delphinapterus leucas*) [36] (Fig. 3A).

We also evaluated the phylogenetic placement of *Acinetobacter* detected in MD 80228 based on both 16S rRNA and *rpo-*β sequences. The number of *rpo-*β sequences for *Acinetobacter* in the database is limited. However, we demonstrated that the MD 80228 transcript-tag clustered with those of *Acinetobacter* (Fig. 3D) [37]. Moreover, based on 16S rRNA, we determined that the *Acinetobacter* species identified in the MD 80228 cDNA library was distinct from all known *Acinetobacter* and was most closely affiliated with *Acinetobacter schindleri* (Fig. 3C).

**Figure 4:**
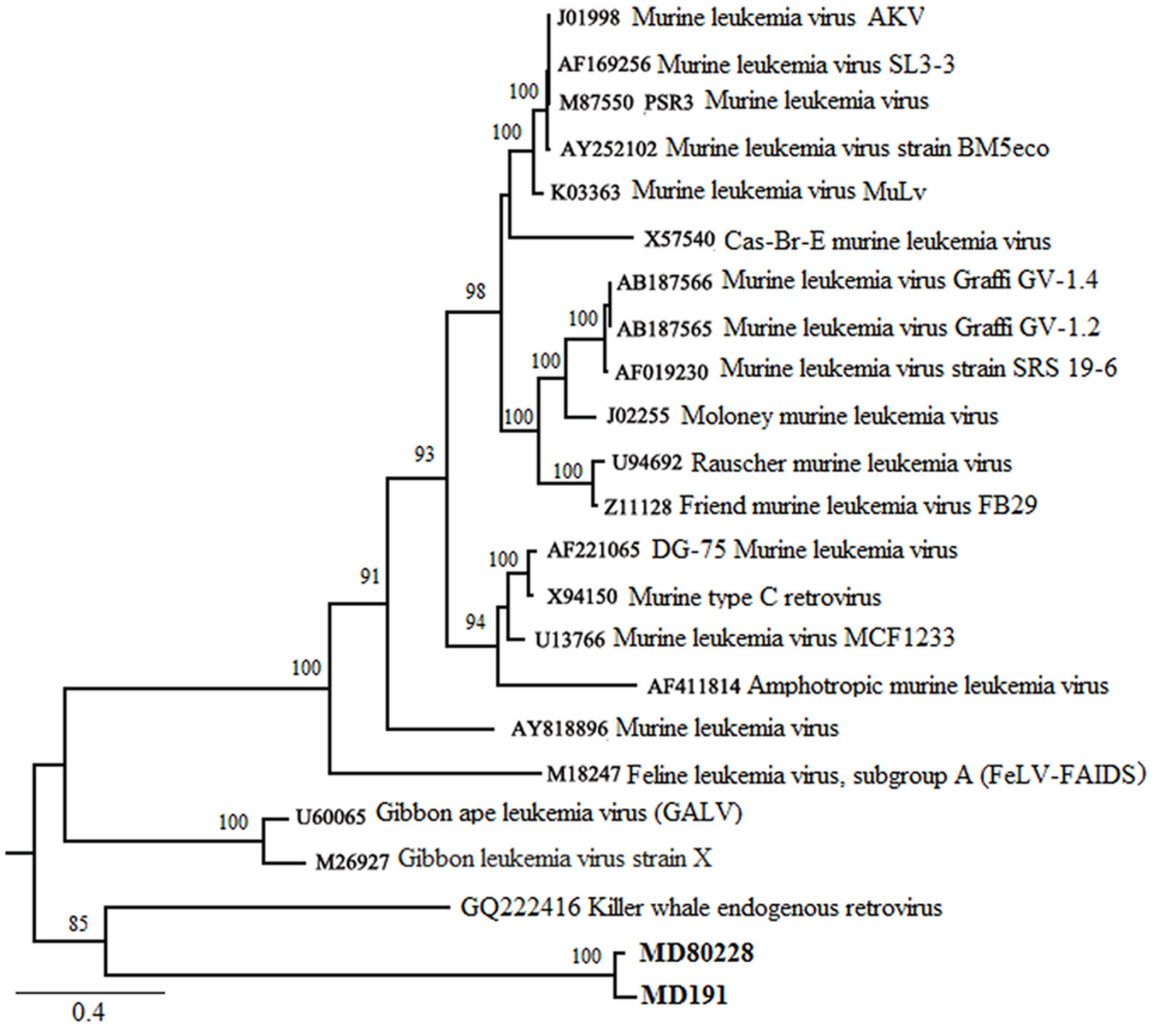
Maximum likelihood tree inferred from the partial nucleotide sequence data of *env* gene showing the phylogenetic placement of mule deer (MD) retrovirus. The two MD PCR sequences reported in the present study are in bold. GenBank accession numbers of reference viruses are mentioned. Bootstrap support for each node is indicated.

The low representation of viral sequences was not unexpected because viruses causing acute infections should be difficult to detect in healthy animals. Retroviruses integrate into the host genome as part of their replication cycle, thus transcription of viral genes can be persistent in infected animals. Overall four transcript-tags were assigned to gamma retroviruses of the family *Retroviridae*. Based on the transcript-tag from the MD Bonner-pool and an upstream region that is conserved in gamma retroviruses, PCR fragments were amplified and sequenced from MD 191 cDNA, which was used in the MD Bonner-pool, and from genomic DNA of MD 80228. These sequences were compared to other gamma retrovirus sequences using maximum likelihood methods (Fig. 4). The mule deer gamma retrovirus forms a distinct clade within the gamma retroviruses, which has many well-described members of primate, murine, and feline origin. A newly described gamma retrovirus from killer whale (*Orcinus orca*) [38] is the closest relative of this mule deer retrovirus. The killer whale virus was described as an endogenous retrovirus based on its finding in various tissues and individuals. However, our detection of transcripts to this virus in only three of the libraries and the sequence variation in the PCR fragment between genomic (MD 80228) and transcript-derived (MD 191) mule deer samples suggest that both endogenous and exogenous gamma retroviruses might be present.

### Comparison of transcript and 16S rRNA amplicon libraries

As an alternative approach to identifying bacterial microorganisms present in lymph node tissue, we utilized amplicon DNA library sequencing technology. The hypervariable region V6 of the 16S rRNA gene was used because it has been reported to differentiate between many bacterial species [39]. Amplicon libraries of V6 were generated from the 454 cDNA libraries of MD 257, MD 80228, and MD OCT-pool and subjected to multiplex pyrosequencing on a Roche GS FLX sequencer (for properties of amplicon sequencing runs, see Table S1). The V6 amplicon rRNA tags were evaluated using the RDP classifier tool (Table S5).

The assigned bacterial genera cluster in the Gamma- and Beta-proteobacteria, the Actinobacteria and in the order Bacilli. A comparison of the three methods used to detect bacteria in mule deer lymph node samples is shown for MD 257 in Figure 2 and for MD 80228 and MD OCT-pool in Figure S1. *Acinetobacter*, *Burkholderia*, *Corynebacterium*, *Escherichia*, *Providencia*, *Salmonella*, *Pseudomonas*, *Ralstonia*, *Staphylococcus*, *Streptococcus* and *Variovorax* were identified by both amplicon and cDNA sequencing in MD 257, MD 80228, and/or MD OCT-pool (Tables S3 and S5).

Although the overall taxonomic diversity in the V6 rRNA amplicon libraries was lower than that detected in the cDNA transcript libraries, the diversity within bacterial classes was higher. Newly identified genera comprised predominantly environmental soil, sediment and water inhabitants (e.g. *Aeromicrobium* and *Bdellovibrio*), and the potential pathogens *Stenotrophomonas*, *Rhodococcus*, *Rothia*, and *Gardnerella* [40]-[43]. These findings indicate that the V6 rRNA amplicon sequencing technology is a valuable tool in complementing information about the bacterial community in host tissues.

## Discussion

Microbiome profiles of environmental samples and animals have mostly been based on the analysis of genomic DNA [8]–[10], [13], [44], [45]. Further, studies on the microbiomes of humans or animals have been restricted to habitats known to harbor large collections of microorganisms, in particular skin, oral cavity or gut [12]–[15], [46]–[48]. In this study, we sought evidence for viable microorganisms in lymph nodes, an organ hitherto believed to be largely amicrobic in the absence of overt disease [26]–[29]. Our data demonstrate that transcriptional activity of a variety of bacteria and a limited number of archaea and viruses, including novel organisms, can be confirmed in healthy animals using a meta-transcriptomic approach.

In our study, we faced the computational challenge of detecting a rare microbial community in a dominant pool of host genetic material. We utilized transcript-based libraries because there is an amplification of protein-coding sequences during transcription, which increased our detection ability and provided support that the identified microorganisms were viable. Further, the database for protein-coding regions is more extensive than that for non-coding regions for non-reference organisms. Thus, focusing on transcripts should facilitate classification of novel organisms and those without complete genome coverage. Indeed, our study demonstrates that at the moderate sequencing depth employed, there were more assignable sequencing tags to protein-coding regions utilizing cDNA compared to genomic DNA, which consequently increased our ability to detect microbial taxa. In addition, transcriptome sequencing yields bacterial ribosomal RNA, which is highly expressed in metabolically active microorganisms and is well documented as a taxonomic tool for bacteria. Because single protein-coding or rRNA transcript-tags from a putative microorganism were frequently encountered, our confidence in taxonomic assignment increased by employing bioinformatics methods to classify organisms based on both types of transcripts. Amplicon 16S rRNA sequencing increased the sensitivity to detect members of some bacterial classes. However, primer specific methods do not provide as comprehensive a perspective on the microbiota due to a possible amplification bias towards more abundant taxa or those exhibiting higher primer specificity. Therefore, neither our metatranscriptomic nor amplicon sequencing approaches should be considered quantitative. We note that in samples that are highly enriched for actively replicating microbial organisms, such as environmental samples or gastrointestinal tract specimens, cDNA-based approaches can yield an abundance of small RNA produced by complex microbial communities, which can facilitate studies on microbial ecology but be less useful for identification of individual microbes [49]. In addition established metagenomics or metatranscriptomic [11], [50], [51] approaches that utilize sample fractionation methods for microbial enrichment will likely provide a more comprehensive profile of the community structure. These methods were not applicable to our samples, which included phagocytized microorganisms and viable microbes that were not robustly proliferating. Nevertheless, as deeper sequencing of cDNA libraries using newer high-throughput sequencing methods becomes more accessible, it could complement the Roche-454 pyrosequencing data, potentially covering the entire viable microbial community.

Our study confirms that there are viable microorganisms in intact lymph nodes of apparently healthy mule deer. In the analyzed samples, we identified members of all bacterial phyla, as well as archaea, a DNA virus and a Retrovirus. The bacteria were representative of organisms that are commensal to mule deer and to their external environment. For example, we detected the common rumen and intestine dwellers, *Ruminococcus* and *Abiotrophia*, based on transcript- and rRNA-tags, respectively, in most libraries, indicating that commensal gut and mucosal microorganisms may routinely be sampled in secondary lymphoid tissue, presumably from transient bacteremia. *Streptomyces* was the most common soil dwelling bacteria identified. Of interest, *Legionella*, which is found near hot springs, was identified only in an individual mule deer from the Yellowstone region. The finding of a considerable number of archaeal transcripts in MD OCT-pool and MD Bonner-pool libraries implies that members of this domain of life are likely present in mule deer habitats or resident in mule deer gastrointestinal tracts, as has been recently documented in humans [52]. Correspondingly, environmental bacteria identified in healthy deer lymph nodes may reflect the animal's habitat.

Few viruses were identified with our analysis methods. This could represent the paucity of viruses in healthy animals. However, viral detection may be more difficult than bacterial identification using this technology in part due to extensive sequence diversity among viruses in the same family. For example, we were only able to detect the gamma retrovirus because a transcript was present which was homologous to a conserved region of the viral *env* gene, and the cervid poxvirus was detected because sequence data for this virus was present in the database. Other persistent viruses, such as herpes viruses (for which we detected a single transcript), would be expected to be present in some animals. However, detection of latent herpes virus infection may be difficult because protein-coding transcript levels are low and latent viruses express non-coding RNA [53]. In addition, viral detection can be compromised if viral sequence tags were misassigned to the host organism because of homology of viral and host genes. Thus, many virus tags might be found among the host transcripts or in the not-assigned or no-hits groups of the MEGAN analysis, which together comprise nearly half of the total sequenced transcript-tags of our data.

In addition to our finding of a novel gamma retrovirus, we also identified new species of *Helicobacter* and *Acinetobacter*. Phylogenetic evaluation of *Helicobacter* transcripts and 16S rRNA from the MD 257 cDNA library placed this new organism in the *Helicobacter pylori/Helicobacter acinonychis/Helicobacter cetorum* complex. All members of this complex have been associated with gastritis and peptic ulcer disease in humans and animals [36], [54]–[56]. Our detection of this bacterium in only one animal suggests that this *Helicobacter* is not a mule deer commensal. Of interest in this respect is the high incidence of *H. pylori* infections and gastric ulcers in American Indian populations from the same geographical area in central Montana [57]. *Acinetobacter* and *Pseudomonas* were identified in MD 80228 libraries based on all detection methods used (cDNA transcripts for protein-coding and rRNA, and amplicon rRNA). Phylogenetic evaluation of *Acinetobacter* transcripts and 16S rRNA from the MD 80228 cDNA library placed the respective reads in close relationship to *Acinetobacter schindleri. Acinetobacter* species are important environmental organisms, however they also are notable pathogens. In particular, *Acinetobacter schindleri* infections appear to be increasing in prevalence in hospitalized patients [37], [58]. Therefore, both of the newly identified bacteria are potential mule deer pathogens.

In conclusion, our study demonstrates that endemic microbiota can be detected in lymph nodes of healthy animals using meta-transcriptomic approaches. These results suggest that meta-transcriptomic analyses of secondary lymphoid organs could be valuable in monitoring endemic infections in wildlife or livestock as well as in detecting novel infectious organisms with the potential for causing emerging zoonotic or epizootic infectious diseases. Further, these studies have the potential to cast new light on the diversity of life within and among individuals.

## Materials and Methods

### Lymph node collection

Retropharyngeal lymph nodes were obtained from a total of seventeen individual Montana mule deer that were presented by hunters to check stations approximately 5 hr (range 2–11 hr) of being shot. Because our samples were obtained from legally killed animals, the study is exempt from Montana State University guidelines governing animal experimentation. Lymph nodes were dissected from animals with sterile scalpel and forceps, and rinsed in 70% ethanol. After dissection from the animal, the lymph nodes were either frozen directly or stored in RNAlater (Applied Biosystems, Ambion, CA) until further processing.

Lymph node tissue was taken from mule deer in several geographical regions. The Bonner pool consisted of tissue from four mule deer (167, 191, 196, 200) from a Montana region north of Interstate 90 in proximity to the town Bonner. The OCT-pool (353, 366, 369, 371, 373, 375, 376, 389) consisted of eight animals from an area in the northwest of Montana defined by the towns Olney, Canoe Gulch, and Thompson Falls. Five mule deer (MD 257, MD 72360, MD 80228, MD 84709, and MD 84730) from different regions were analyzed individually (Fig. S3).

### Preparation of genomic DNA, total RNA, poly(A)^+^RNA and cDNA

Lymph node tissue cores were dissected into small pieces and further disrupted, lysed and homogenized using a TissueLyser with steel beads (Qiagen, Germany). Genomic DNA was isolated from lymph nodes of four individual Mule deer (MD 72360, MD 80228, MD 84709, and MD 84730) using either the Genomic DNA Buffer Set with 20/G Genomic-tips (Qiagen, Germany) or the AllPrep DNA/RNA Mini Kit (Qiagen, Germany). Total RNA was isolated using the RNAqueous-Midi Kit (Applied Biosystems, Ambion, CA). For the Bonner- and OCT-pools, equal quantities of total RNA from lymph nodes of four or eight individual Mule Deer, respectively, were combined. Poly(A)^+^RNA was enriched from total RNA using the MicroPoly(A) Purist Kit (Applied Biosystems, Ambion, CA). Poly(A)^+^RNA (0.9–5.0 µg each) was used for cDNA synthesis (Just cDNA Double-Stranded cDNA Synthesis Kit, Stratagene, CA) after elimination of residual contaminating genomic DNA using the Turbo DNA-free Kit (Applied Biosystems, Ambion, CA). In one case we explored an alternative empirical approach to enrich for rare microbial transcripts, using total RNA of the MD OCT-pool. Reverse transcription and amplification of cDNA was done as described by Cheung and coworkers [59] and included a normalization step, which effectively decreased over- expressed reads. The data resulting from this approach are included in the MD OCT-pool data.

### Roche-454 GS FLX pyrosequencing

Up to 5.0 µg of cDNA or genomic DNA was subjected directly to preparation of 454-DNA libraries and subsequently to pyrosequencing without any prior PCR or cloning steps. Library preparation and pyrosequencing were performed as described previously [60] on a Roche GS20 sequencer FLX (Roche Applied Sciences/454 Life Sciences, Branford, CT), producing sets of RNA-tags or DNA-tags, respectively. The runs were performed on either quarter or half plates, resulting in read numbers between 10,673 and 176,878 and base numbers in the range of 1,411,420 to 41,066,808. The MD OCT-pool cDNA library was run twice due to low read and base numbers of the first run, and the transcript-tags of these two runs and of the run following the normalization approach (see above) were combined for all subsequent data analysis. Sequences are deposited to the Sequence Read Archive (in progress).

### Data analysis

The data of individual 454 runs (and the compilation of normal and normalized MD OCT-pool data) was compared against the NCBI non-redundant protein database (BLASTX-nr) with an e-value of 1e^-4^ to identify transcript RNA-derived tags. To filter repetitive elements, RepeatMasker (http://www.repeatmasker.org) was used to scan the mule deer sequences, with the latest version of Repbase 13.04 [61]. The output files were analyzed with the program MEGAN [32] version 3.7.2.

The 16S ribosomal RNA content of the cDNA pyrosequencing reads was analyzed by comparison to the ribosomal database of the Ribosomal Database Project (RDP) version 10 (http://rdp.cme.msu.edu/) [34]. The selected output reads were classified by the RDP Classifier tool (Naïve Bayesian rRNA Classifier Version 2.0) using the Taxonomic Outline of the Bacteria and Archaea, release 7.8, for the setup of the taxonomical hierarchy [35]. The output files were analyzed with MEGAN version 3.7.2 [32]. For the MD OCT-pool, the combined data of three individual 454 runs was used.

### Virus amplification

The cDNA from MD 191, which was used in the MD Bonner pool, and genomic DNA from MD 80228 were subjected to PCR using forward primer 5-ATGTGGGGGAGTTGATTCTTTTTA and reverse primer 5-CTGCGCCTGAGTGGTCTACATA. PCR conditions were 40 cycles of 95°C for 30 sec, 56°C for 30 sec and 72°C for 90 sec. Fragments were gel isolated, cloned using the Stratagene PCR cloning kit (Stratagene, La Jolla, CA) and Sanger sequenced.

### Phylogenetic analyses

Partial nucleotide sequences of 16S rRNA and *rpo-*β for *Helicobacter* and *Acinetobacter* and of *flg*K, GDP-D-mannose dehydratase and UDP-3-O-[3-hydroxymyristoyl] glucosamine N-acyltransferase for *Helicobacter* from cDNA sequencing, and of *env* gene for the retrovirus from a PCR product were aligned with the respective homologous sequences available in GenBank using the MEGA version 4 [56] software. The appropriate nucleotide substitution model for each data set was selected by the Akaike information criterion implemented in the Modeltest version 3.7 [62], and maximum likelihood (ML) trees were reconstructed using PhyML version 2.4.4 [63]. Using the same program (PhyML) nodal supports were estimated with 100 bootstrap replicates. The trees were visualized in FigTree version 1.2.2 (http://tree.bio.ed.ac.uk/software/figtree/).

### Multiplex Amplicon Sequencing (Roche-454)

Fusion-primers were designed including the sequences of the 454-Amplicon DNA library specific primers A and B, respectively, (GS FLX Amplicon DNA Library Preparation Method Manual, www.roche-applied-science.com), 4-base barcode sequences for identifying amplicon products derived from mule deer specimen MD 257, MD OCT-pool, and MD 80228 (TGCA, ACGT, and CGAT, respectively), and the “universal” V6-specific PCR primer sequences V6F: 5′ TCGATGCAACGCGAAGAA 3′ and V6R: 5′ ACATTTCACAACACGAGCTGACGA 3′ (designed to conserved regions flanking V6 based on comparison of 110 bacterial DNA sequences [39]).

The MD 257 template for amplicon generation was based on the total RNA fraction depleted of poly(A)^+^RNA (see “Preparation of genomic DNA, total RNA, poly(A)+RNA and cDNA”). The supernatant was cleared of small RNA molecules using the MEGAclear Kit (Applied Biosystems, Ambion, CA) and depleted of host ribosomal RNA performing two cycles of the MICROB*Enrich* (Applied Biosystems, Ambion, CA) protocol. Subsequent depletion of bacterial ribosomal RNA yielded an RNA sample enriched for bacterial transcripts (MICROB*Express*, Applied Biosystems, Ambion, CA), which was subjected to cDNA synthesis (Just cDNA Double-Stranded cDNA Synthesis Kit, Stratagene, CA) after elimination of residual contaminating genomic DNA using the Turbo DNA-free Kit (Applied Biosystems, Ambion, CA).

Either cDNA derived from RNA enriched for non-polyadenylated bacterial mRNA (MD 257) or cDNA sequencing library samples derived from reverse transcribed poly(A)^+^RNA (for MD OCT-pool and MD 80228) were used as templates for the generation of 16S rRNA V6 hypervariable region-specific amplicons using the FastStart High Fidelity PCR System (Roche, Switzerland). PCR conditions were 50 cycles of 94°C for 30 sec, 55°C for 45 sec and 72°C for 45 sec. The yielded amplicon products were purified using AMPure, and the resulting individual amplicon DNA libraries were clonally amplified by multiplex emulsion PCR followed by sequencing using the GS FLX pyrosequencing platform. The sequencing output data were computationally divided into subsets according to the barcodes (and the corresponding mule deer sample) and the primers A or B.

## Supporting Information

Figure S1 Comparative MEGAN analysis of (A) MD 80228 and (B) MD OCT-pool transcript-tags analyzed by comparison to the protein database (red) and the ribosomal database (blue), and of amplicon 16S rRNA-tags compared to the ribosomal database (green). Bit score cutoff for the protein database comparison was set at 50, and confidence cutoffs for the ribosomal database comparisons were set at 80% and 80%, respectively.
(0.45 MB DOC)

Figure S2 Maximum likelihood trees showing the phylogenetic affiliation of protein-coding transcripts obtained from 454 sequencing with *Helicobacter* reference sequences from GenBank. (A) *Helicobacter* FlgK, (B) *Helicobacter* GDP-D-mannose dehydratase, (C) *Helicobacter* UDP-3-O-[3-hydroxymyristoyl] glucosamine N-acyltransferase.
(0.18 MB DOC)

Figure S3 Map of Montana, USA, depicting the geographical distribution of the mule deer specimen.
(8.14 MB DOC)

Table S1 Properties of Roche-454 GS FLX sequencing runs.
(0.42 MB DOC)

Table S2 Numbers of cDNA transcript-tags and genomic DNA-tags of seven and four mule deer specimen, respectively, assigned to major taxonomic nodes by MEGAN comparison (bit score cutoff set at 50).
(0.30 MB DOC)

Table S3 Numbers of transcript-tags assigned to bacterial taxa by MEGAN comparison for seven mule deer lymph node specimen.
(0.73 MB DOC)

Table S4 Bacterial taxonomic profiles of seven mule deer specimen determined by comparison of cDNA libraries-derived rRNA-tags to the ribosomal database.
(0.51 MB DOC)

Table S5 Bacterial taxonomic profiles of mule deer specimen MD 257, MD 80228, and MD OCT-pool determined by comparison of amplicon 16S rRNA-tags to the ribosomal database.
(0.60 MB DOC)
