# Supplementary material for: Nodeomics: Pathogen Detection in Vertebrate Lymph Nodes Using Meta-Transcriptomics
Source: PLoS One. 2010 Oct 18;5(10):e13432. doi: 10.1371/journal.pone.0013432 (PMC2956653; doi:10.1371/journal.pone.0013432)

**SUPPORTING INFORMATION**

**Figure S1:** Comparative MEGAN analysis of (A) MD 80228 and (B) MD OCT-pool transcript-tags analyzed by comparison to the protein database (red) and the ribosomal database (blue), and of amplicon 16S rRNA-tags compared to the ribosomal database (green). Bit score cutoff for the protein database comparison was set at 50, and confidence cutoffs for the ribosomal database comparisons were set at 80% and 80%, respectively.

**(A)**


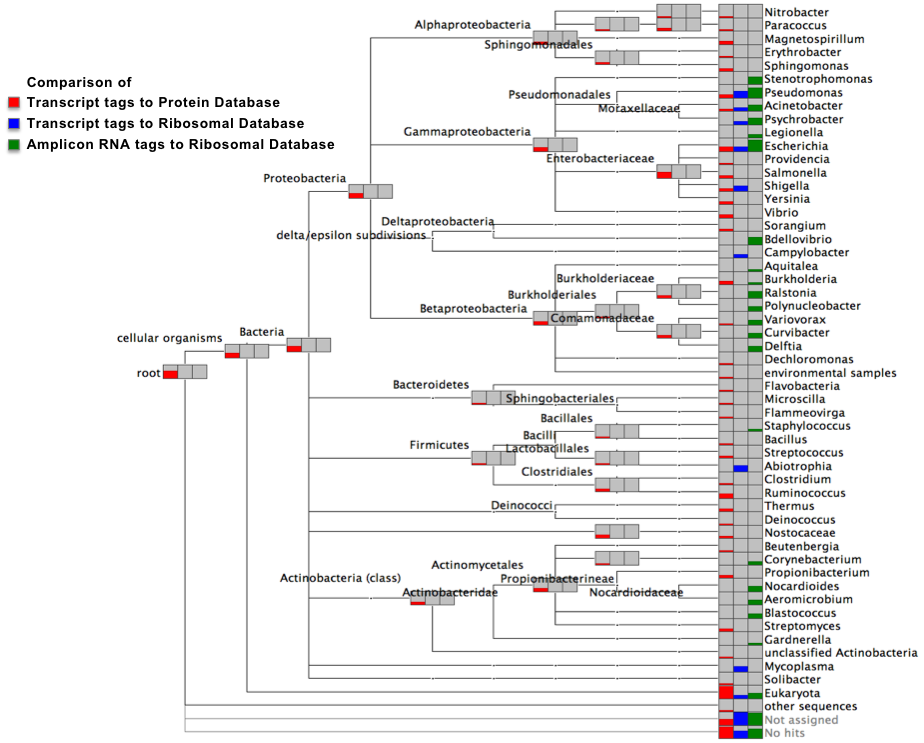


**(B)**


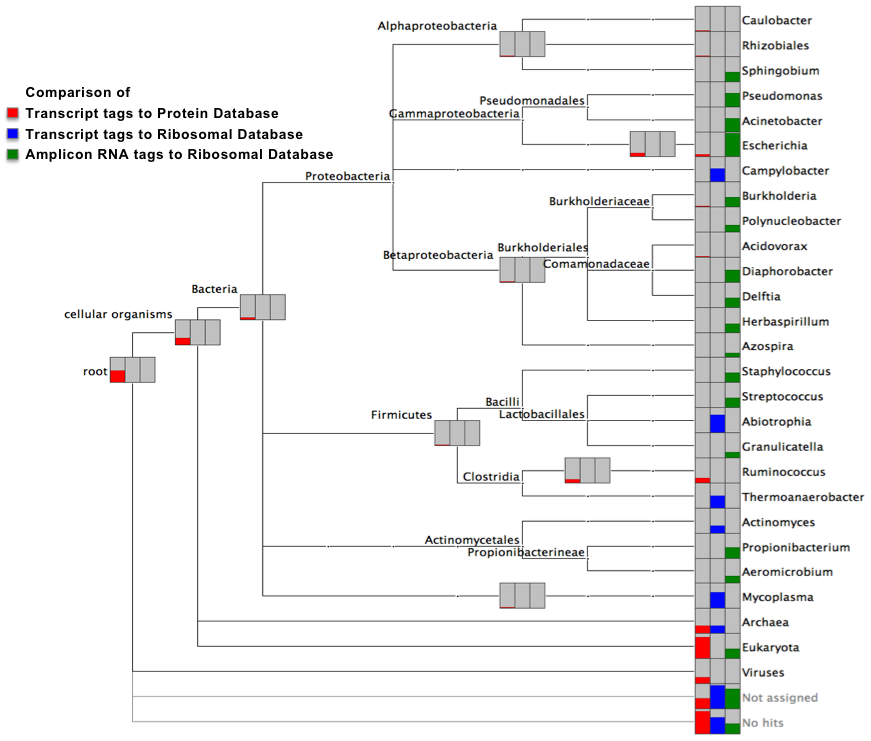

Supplement: Figure S1 — Comparative MEGAN analysis of (A) MD 80228 and (B) MD OCT-pool transcript-tags analyzed by comparison to the protein database (red) and the ribosomal database (blue), and of amplicon 16S rRNA-tags compared to the ribosomal database (green). Bit score cutoff for the protein database comparison was set at 50, and confidence cutoffs for the ribosomal database comparisons were set at 80% and 80%, respectively. (0.45 MB DOC) [file pone.0013432.s001.doc]
