# Supplementary material for: Nodeomics: Pathogen Detection in Vertebrate Lymph Nodes Using Meta-Transcriptomics
Source: PLoS One. 2010 Oct 18;5(10):e13432. doi: 10.1371/journal.pone.0013432 (PMC2956653; doi:10.1371/journal.pone.0013432)
Supplement: Figure S2 — Maximum likelihood trees showing the phylogenetic affiliation of protein-coding transcripts obtained from 454 sequencing with Helicobacter reference sequences from GenBank. (A) Helicobacter FlgK, (B) Helicobacter GDP-D-mannose dehydratase, (C) Helicobacter UDP-3-O-[3-hydroxymyristoyl] glucosamine N-acyltransferase. (0.18 MB DOC) [file pone.0013432.s002.doc]

**Figure S2:** Maximum likelihood trees showing the phylogenetic affiliation of protein-coding transcripts obtained from 454 sequencing with *Helicobacter* reference sequences from GenBank. (A) *Helicobacter* FlgK, (B) *Helicobacter* GDP-D-mannose dehydratase, (C) *Helicobacter* UDP-3-O-[3-hydroxymyristoyl] glucosamine N-acyltransferase.

**(A)**

**
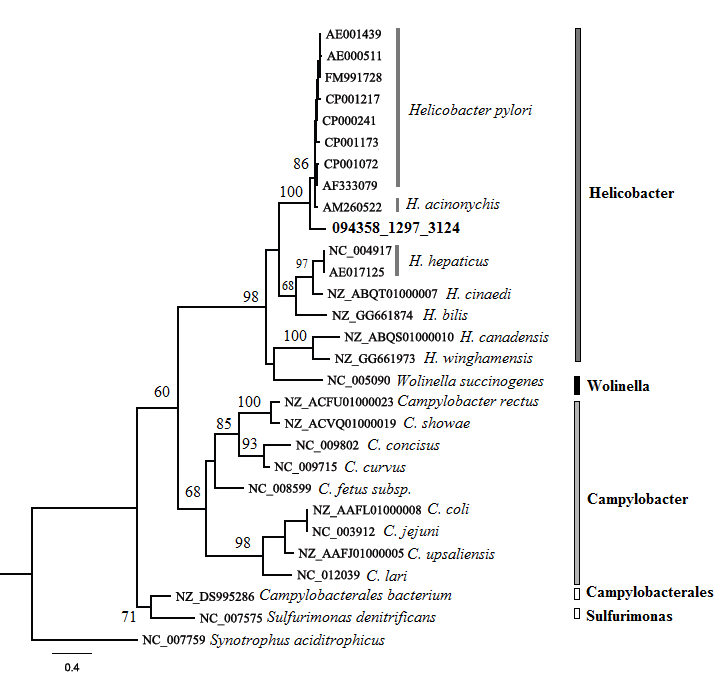
**

**(B)**

**
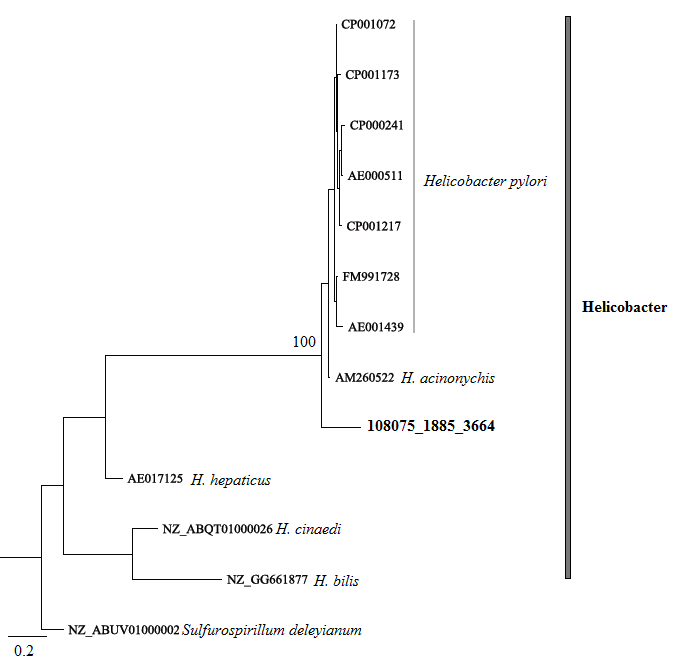
**

**(C)**

**
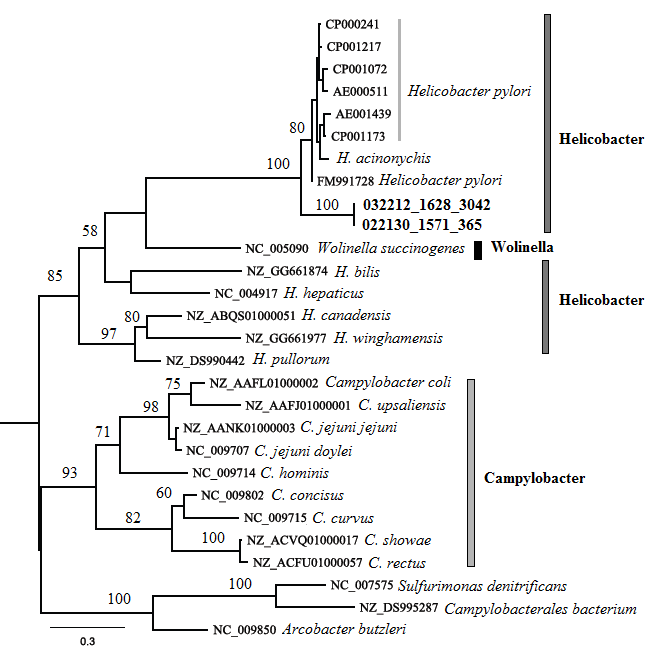
**
