# Supplementary figures and images for: Nodeomics: Pathogen Detection in Vertebrate Lymph Nodes Using Meta-Transcriptomics
Source: PLoS One. 2010 Oct 18;5(10):e13432. doi: 10.1371/journal.pone.0013432 (PMC2956653; doi:10.1371/journal.pone.0013432)

**Figure S3:** Map of Montana, USA, depicting the geographical distribution of the mule deer specimen.


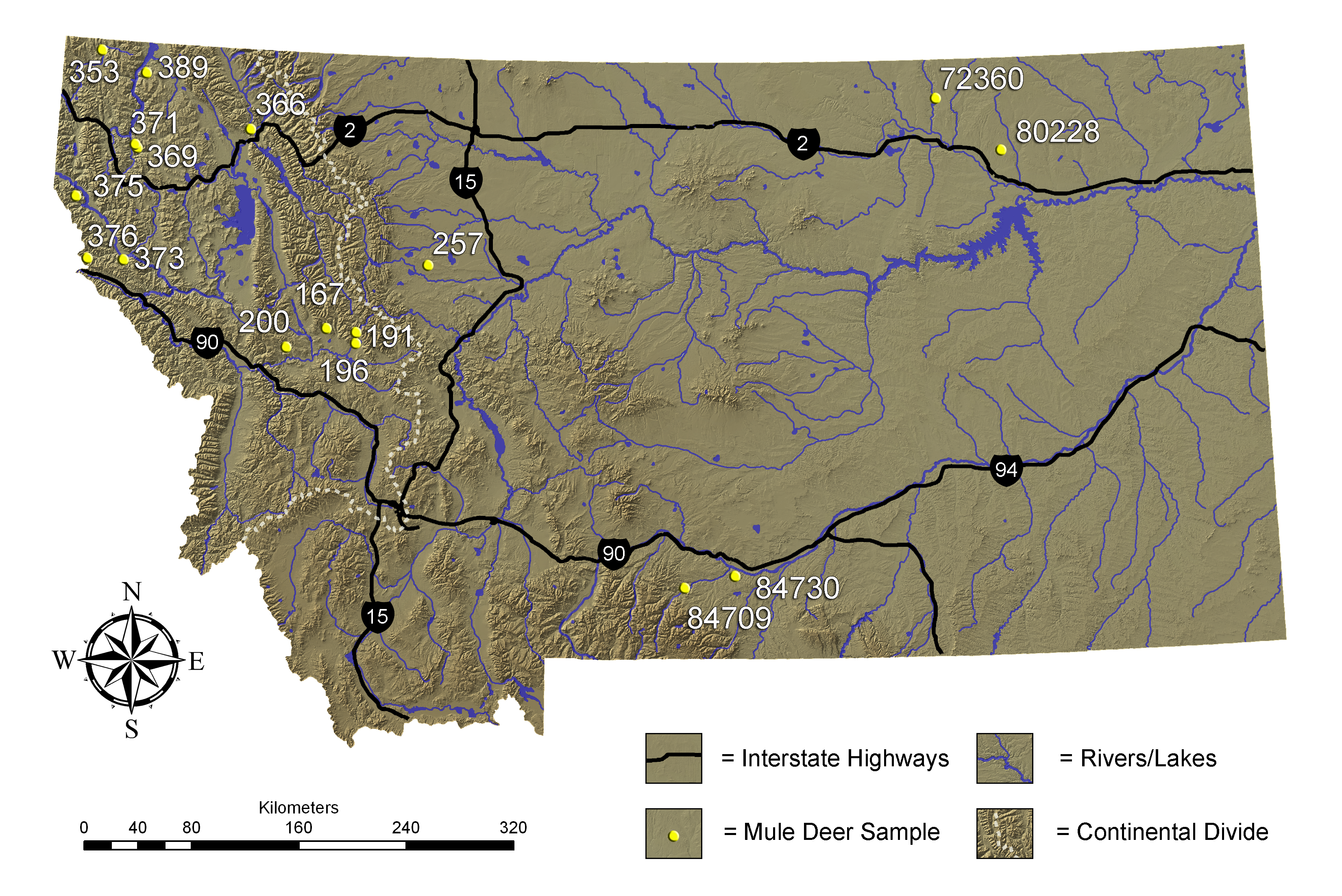

Supplement: Figure S3 — Map of Montana, USA, depicting the geographical distribution of the mule deer specimen. (8.14 MB DOC) [file pone.0013432.s003.doc]

**Table S1:** Properties of Roche-454 GS FLX sequencing runs.

**
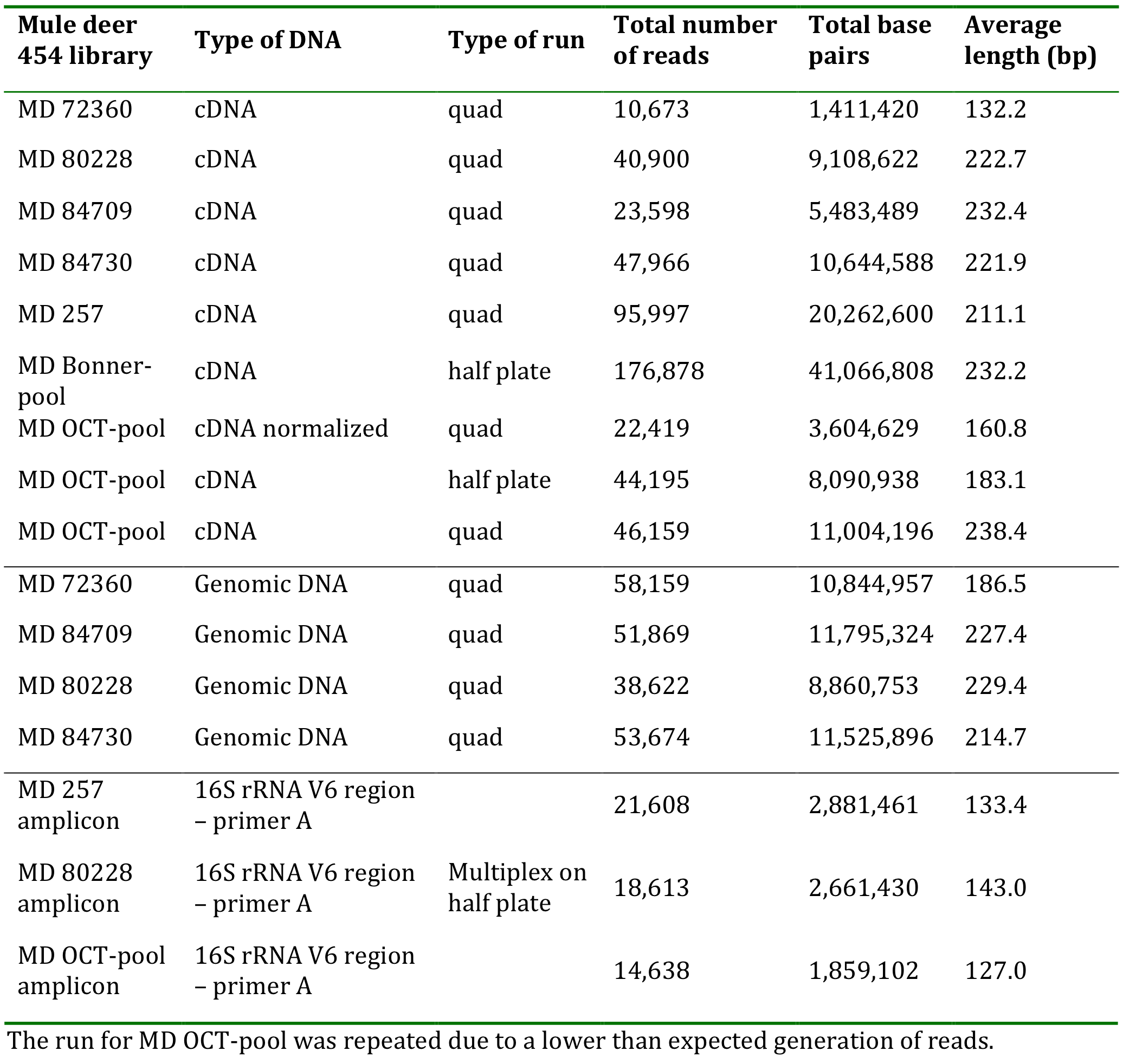
**

Supplement: Table S1 — Properties of Roche-454 GS FLX sequencing runs. (0.42 MB DOC) [file pone.0013432.s004.doc]

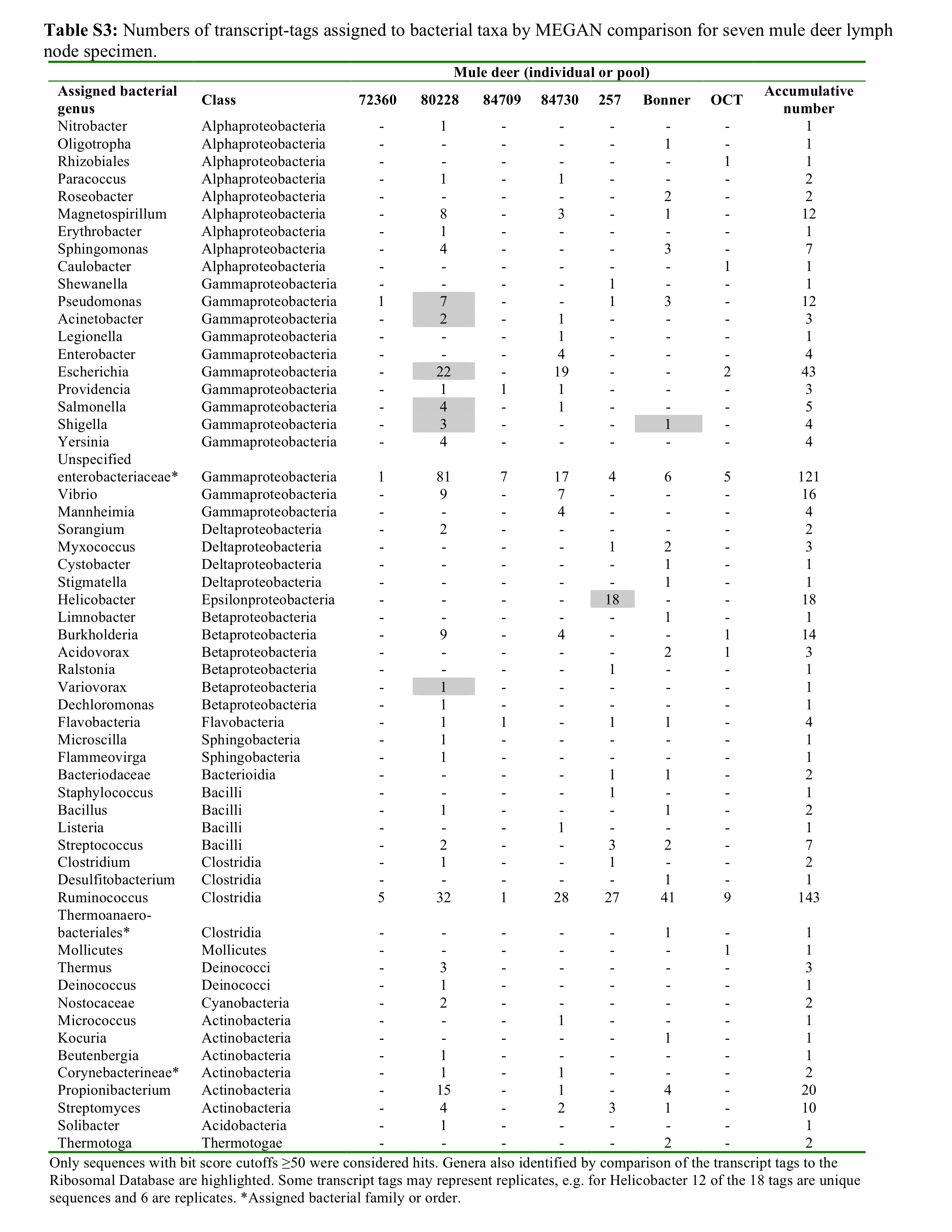

Supplement: Table S3 — Numbers of transcript-tags assigned to bacterial taxa by MEGAN comparison for seven mule deer lymph node specimen. (0.73 MB DOC) [file pone.0013432.s006.doc]
