# Supplementary material for: Nodeomics: Pathogen Detection in Vertebrate Lymph Nodes Using Meta-Transcriptomics
Source: PLoS One. 2010 Oct 18;5(10):e13432. doi: 10.1371/journal.pone.0013432 (PMC2956653; doi:10.1371/journal.pone.0013432)

**Table S2:** Numbers of cDNA transcript-tags and genomic DNA-tags of seven and four mule deer specimen, respectively, assigned to major taxonomic nodes by MEGAN comparison (bit score cutoff set at 50).


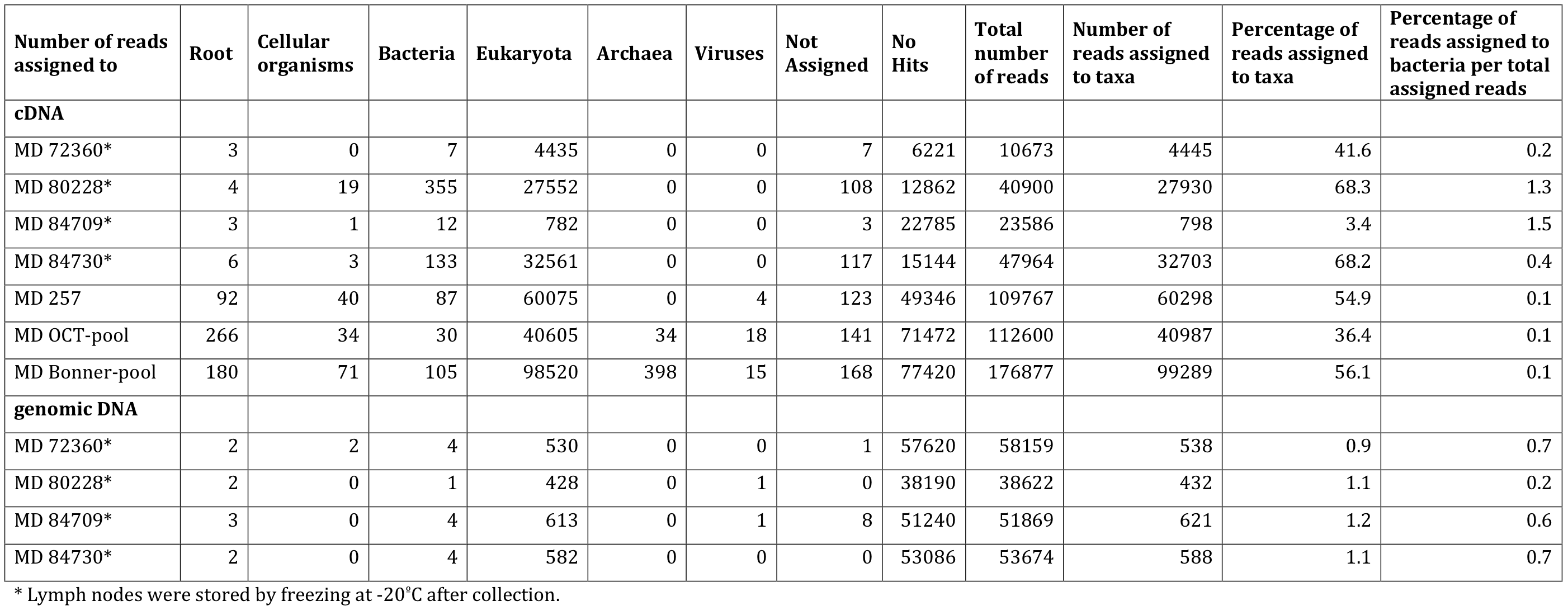

Supplement: Table S2 — Numbers of cDNA transcript-tags and genomic DNA-tags of seven and four mule deer specimen, respectively, assigned to major taxonomic nodes by MEGAN comparison (bit score cutoff set at 50). (0.30 MB DOC) [file pone.0013432.s005.doc]
