# Supplementary material for: Nodeomics: Pathogen Detection in Vertebrate Lymph Nodes Using Meta-Transcriptomics
Source: PLoS One. 2010 Oct 18;5(10):e13432. doi: 10.1371/journal.pone.0013432 (PMC2956653; doi:10.1371/journal.pone.0013432)

**Table S4:** Bacterial taxonomic profiles of seven mule deer specimen determined by comparison of cDNA libraries-derived rRNA-tags to the ribosomal database.


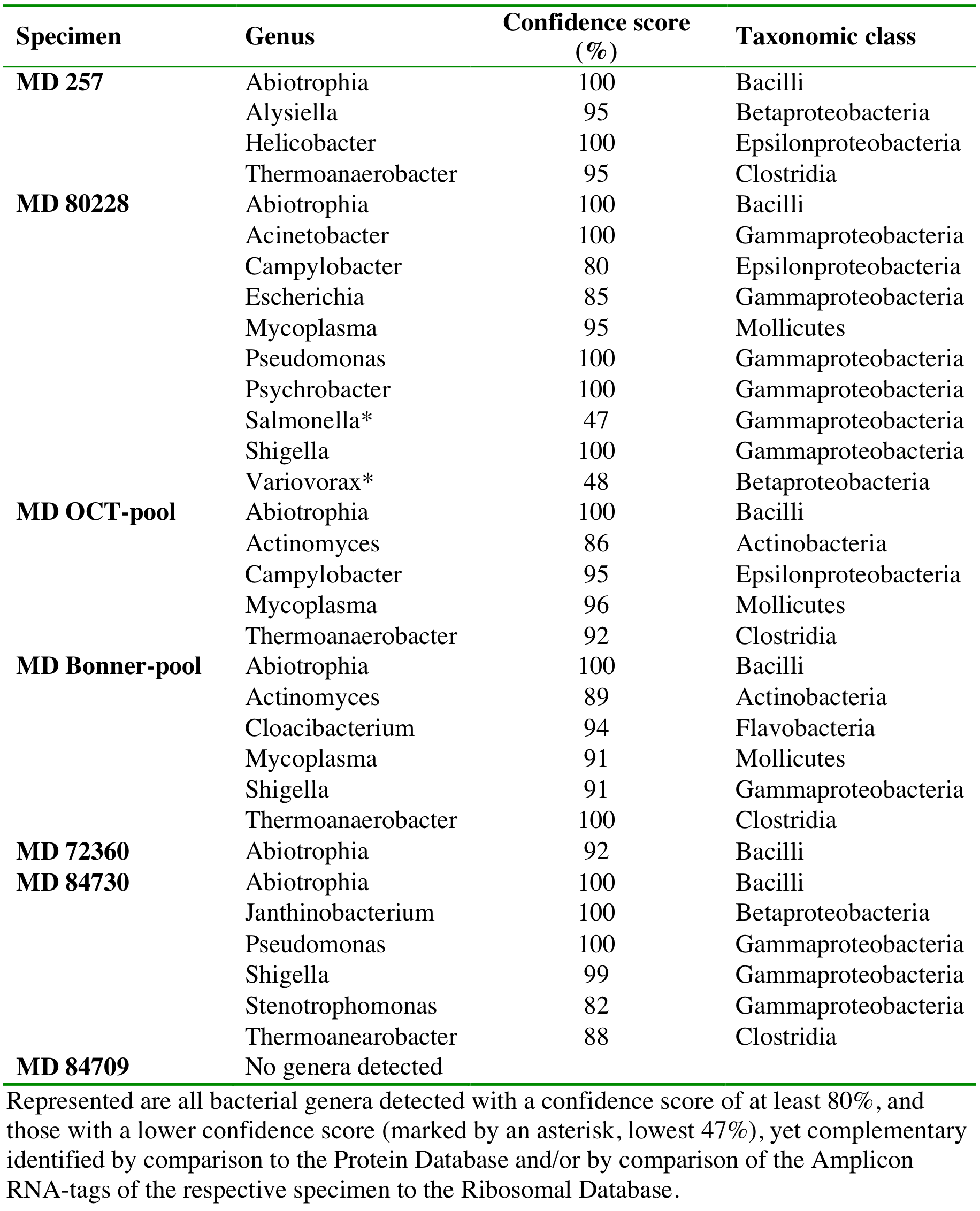

Supplement: Table S4 — Bacterial taxonomic profiles of seven mule deer specimen determined by comparison of cDNA libraries-derived rRNA-tags to the ribosomal database. (0.51 MB DOC) [file pone.0013432.s007.doc]
