# Supplementary material for: Nodeomics: Pathogen Detection in Vertebrate Lymph Nodes Using Meta-Transcriptomics
Source: PLoS One. 2010 Oct 18;5(10):e13432. doi: 10.1371/journal.pone.0013432 (PMC2956653; doi:10.1371/journal.pone.0013432)
Supplement: Table S5 — Bacterial taxonomic profiles of mule deer specimen MD 257, MD 80228, and MD OCT-pool determined by comparison of amplicon 16S rRNA-tags to the ribosomal database. (0.60 MB DOC) [file pone.0013432.s008.doc]

**Table S5:** Bacterial taxonomic profiles of mule deer specimen MD 257, MD 80228, and MD OCT-pool determined by comparison of amplicon 16S rRNA-tags to the ribosomal database.

**
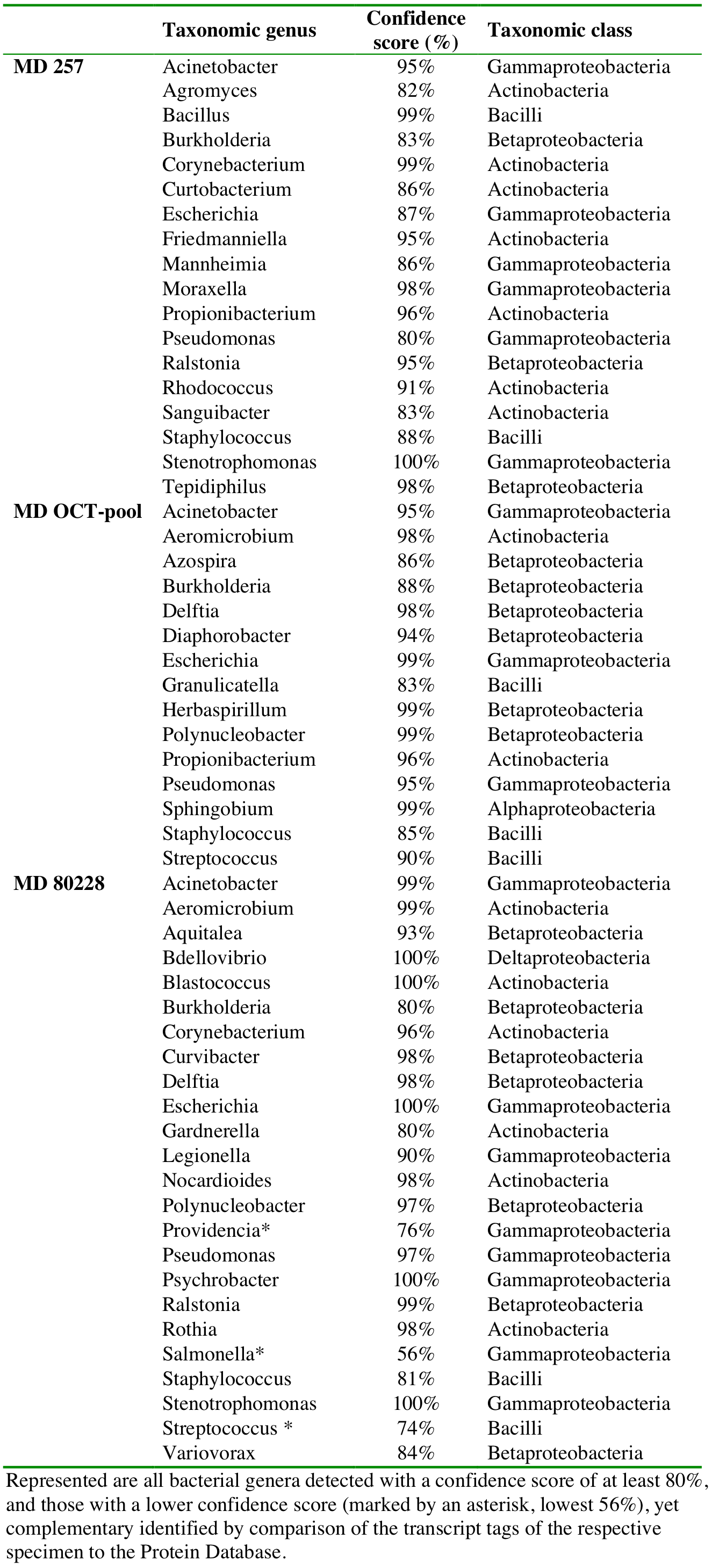
**
